# Early surgical intervention is critical for strangulated Richter’s hernia

**DOI:** 10.1093/jscr/rjae642

**Published:** 2024-10-11

**Authors:** Christopher R Smith, Michail Chatzikonstantinou

**Affiliations:** Department of General Surgery, Lewisham and Greenwich NHS Trust, University Hospital Lewisham, Lewisham High St., London SE13 6LH, United Kingdom; Department of General Surgery, Lewisham and Greenwich NHS Trust, University Hospital Lewisham, Lewisham High St., London SE13 6LH, United Kingdom

**Keywords:** inguinal hernia, abdominal hernia, emergency general surgery

## Abstract

Richter’s hernia is a rare but serious surgical emergency involving the entrapment or strangulation of part of the bowel’s circumference in the hernial orifice, often without causing complete luminal obstruction. This case report describes a man in his 70s presenting with a 3-day history of abdominal pain and vomiting, despite normal bowel movements. Blood results revealed raised inflammatory markers, and computed tomography imaging suggested small bowel obstruction due to an incarcerated left inguinal hernia. Emergency surgery confirmed a Richter’s hernia with a strangulated but viable bowel loop. Early surgical intervention led to a positive outcome. This case underscores the importance of high suspicion, early referral, and timely imaging in managing Richter’s hernia to prevent severe complications, such as gangrene and perforation. Despite the absence of obstructive symptoms, early surgical intervention is critical when there is clinical concern regarding strangulation.

## Introduction

First comprehensively described by August Gottlob Richter in 1785 [1], Richter’s hernia is an abdominal hernia where only a portion of the bowel’s circumference is entrapped or strangulated in the hernial orifice [2]. This rare type of hernia can cause rapid ischaemia without causing intestinal obstruction. It can occur at any typical hernial site but is most common in small hernial defects with firm margins, such as the femoral ring and inguinal canal [3]. Early surgical intervention is crucial for successful management and recognizing its clinical features and potentially misleading presentation is essential.

## Case report

A man in his 70s presented to the Emergency Department overnight with a 3-day history of abdominal pain and vomiting, though he had been opening his bowels normally. His medical history includes chronic obstructive pulmonary disease (COPD) and benign prostatic hyperplasia (BPH), with no previous history of abdominal surgery. He is a current smoker and consumes alcohol occasionally. He has no significant family history.

On admission, he was hypertensive (161/90 mmHg), tachycardic (113 bpm), but apyrexial. Physical examination revealed a distended, soft, and non-tender abdomen, with bilateral groin swellings indicative of inguinal hernias. The left inguinal hernia was irreducible, while the right was fully reducible. Both inguinal hernia was tender on palpation, and the overlying skin appeared normal.

Blood tests showed raised inflammatory markers (C-reactive protein 68 mg/ml, white blood cell count 19.0 × 10^9^/L, neutrophil count 16.7 × 10^9^/L) and a stage 1 acute kidney injury (serum creatinine 116 μmol/L from 66 μmol/L).

**Figure 1:**
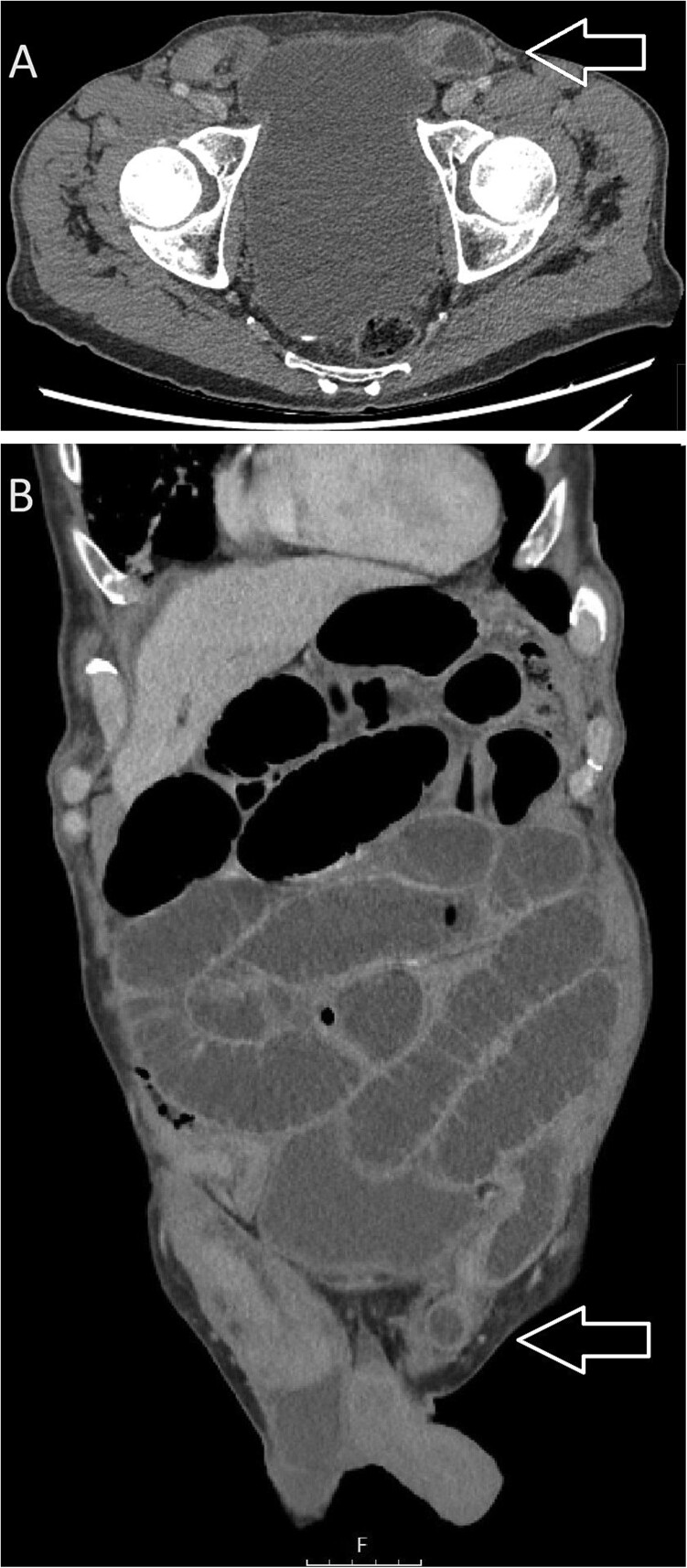
CT images: (A) axial and (B) coronal views suggestive of small bowel obstruction secondary to an obstructed, incarcerated left inguinal hernia. Arrows have been added to the image to mark the site of the left inguinal hernia.

Computed tomography (CT) of the abdomen and pelvis revealed significant dilatation of the small bowel loops, measuring up to 40 mm in diameter, with a sharp transition point at the site of the left inguinal hernia, suggesting small bowel obstruction due to an obstructed, incarcerated left inguinal hernia (see Fig. 1). The CT also identified early pneumonia in the lung bases bilaterally.

Initial management included starting intravenous antibiotics, resuscitating with intravenous fluids, and inserting a large-bore nasogastric tube for decompression, though the patient declined this after a lengthy discussion with the surgical team.

**Figure 2:**
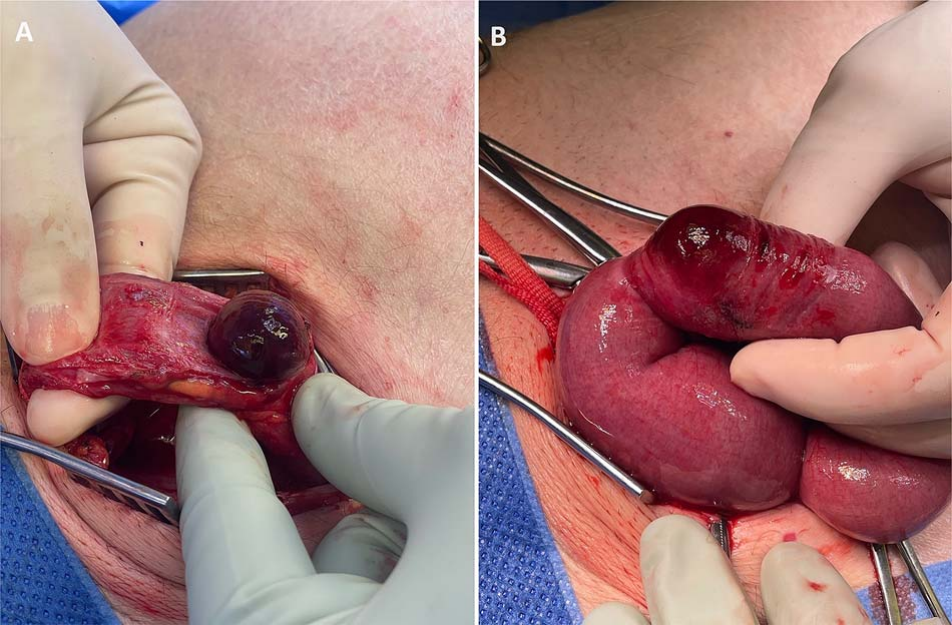
Intra-operative photographs: (A) strangulated small bowel on opening the indirect inguinal hernia sac, and (B) affected small bowel segment once delivered, demonstrating that only the anti-mesenteric portion of small bowel was entrapped within the hernia—findings consistent with a Richter’s hernia.

Following consultation with a consultant surgeon and anaesthetist, the patient underwent an emergency open inguinal hernia repair under spinal anaesthesia. Intra-operative findings revealed a large indirect inguinal hernia containing serous fluid and a strangulated anti-mesenteric portion of a small bowel loop (Richter’s hernia) (see Fig. 2). After applying warm saline, the small bowel loop was deemed viable with good peristalsis. The small bowel was returned to the abdominal cavity, and the proximal end of the hernia sac was closed. The posterior wall of the inguinal canal was plicated, and the deep inguinal ring was reconstructed laterally. Given the bowel’s status and the presence of copious serous fluid, no mesh was placed.

The patient was admitted to the high dependency unit post-operatively and closely observed. He was transferred to the surgical ward on postoperative Day 1 and discharged on postoperative Day 7, after making a full recovery to his normal function. Three days after discharge, he re-presented with a lower urinary tract infection and was discharged with antibiotics.

The patient was re-reviewed 8 weeks post-operatively. Due to a fall, he was re-admitted under the orthopaedic team at the time of his planned general surgery follow-up, so he was reviewed on the surgical ward instead of the outpatient clinic. Upon review, he had recovered well from his hernia surgery with no evidence of inguinal hernia recurrence on examination. He was subsequently discharged from general surgery follow-up.

## Discussion

**Figure 3:**
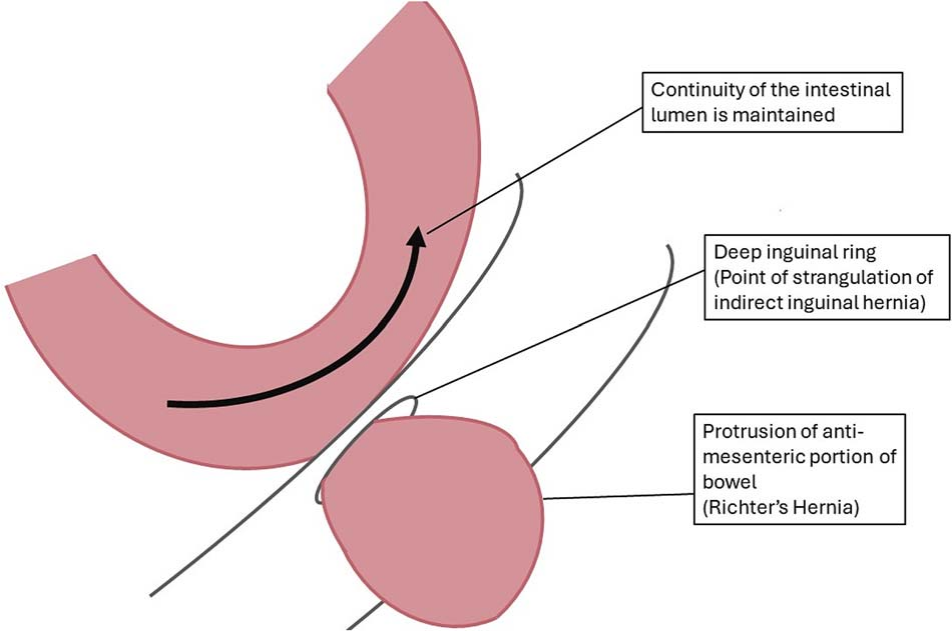
Labelled diagram of strangulated indirect inguinal Richter’s hernia. Created by CR Smith.

Richter’s hernias account for ~5%–15% of all strangulated hernias, typically affecting elderly patients but can occur at any age, including childhood [2]. The mortality rate for Richter’s hernia is estimated at 17% [2]. We present a case of a Richter’s hernia at the deep inguinal ring, manifesting as an irreducible, painful left inguinal hernia (see Fig. 3).

Richter’s hernias often lack symptoms and signs of intestinal obstruction, leading to delayed diagnosis and severe complications, such as bowel gangrene and perforation. Numerous case reports highlight the varied acute presentations of Richter’s hernia, emphasizing the diagnostic difficulty due to the rare occurrence of complete luminal obstruction [4–7]. Chronic incarceration can also occur due to the absence of obstructive symptoms, potentially resulting in an enterocutaneous fistula if untreated [8].

In our case, the CT scan indicated small bowel obstruction at the hernia, likely expediting the decision for emergency surgery—though this is not the case for all Richter’s hernias due to the typical absence of luminal obstruction. Strangulation of the bowel was not identified on imaging but noted intra-operatively. We advocate for early surgical team involvement in cases of acute clinical concern. While an emergency CT scan is a valuable diagnostic tool, it should not delay surgical management when strangulation is highly suspected.

As with all strangulated hernias, Richter’s hernias require immediate surgical intervention. Treatment depends on bowel viability and local contamination, often necessitating bowel resection and repair of the fascial defect, with or without synthetic mesh. In our case, emergency open hernia repair was performed without bowel resection as the bowel was viable, illustrating that urgent surgical intervention can prevent progression to irreversible pathology such as gangrene, which would require bowel resection.

## Lay summary

- Richter’s hernia, although rare, requires prompt surgical intervention to prevent severe complications, such as bowel gangrene and perforation.
- The diagnosis of Richter’s hernia is challenging as patients often present without typical signs and symptoms of bowel obstruction because herniation of only the anti-mesenteric portion of the bowel wall does not usually cause complete luminal obstruction.
- High clinical suspicion, early referral, and timely imaging are essential for effective management. Strangulated Richter’s hernia should be considered in any patient with a severely painful hernia, even in the absence of obstructive symptoms.
